# Cordycepin is a novel chemical suppressor of Epstein-Barr virus replication

**DOI:** 10.18632/oncoscience.110

**Published:** 2014-12-18

**Authors:** Eunhyun Ryu, Myoungki Son, Minjung Lee, Kanghyo Lee, Jae Youl Cho, Sungchan Cho, Suk Kyeong Lee, You Mie Lee, Hyosun Cho, Gi-Ho Sung, Hyojeung Kang

**Affiliations:** ^1^ College of Pharmacy and Institute of microorganisms, Kyungpook National University, Daegu, Republic of Korea; ^2^ Mushroom Research Division, National Institute of Horticultural and Herbal Science, Rural Development Administration, Eumseong, Republic of Korea; ^3^ Department of Genetic Engineering, Sungkyunkwan University, Suwon, Republic of Korea; ^4^ Targeted Medicine Research Center, Korea Research Institute of Bioscience and Biotechnology, Cheongwon, Chungbuk, Republic of Korea; ^5^ Research Institute of Immunobiology, Department of Biomedical Sciences, College of Medicine, The Catholic University of Korea, Seoul, Republic of Korea; ^6^ College of Pharmacy, Duksung Women's University, Seoul, Republic of Korea; ^7^ Institute for Bio-Medical Convergence, International St. Mary's Hospital, College of Medicine, Catholic Kwangdong University, Incheon, Republic of Korea

**Keywords:** Cordycepin, Epstein–Barr virus, gastric carcinoma, antiviral agent

## Abstract

*Cordyceps* species are known to produce numerous active components and are used for diverse medicinal purposes because of their varied physiological activities, including their ability to protect the liver from damage as well as their anticancer, antidepressant, anti-inflammatory, hypoglycemic, antimicrobial effects. Cordycepin, an adenosine derivative, differs from adenosine in that its ribose lacks an oxygen atom at the 3′ position. Several research groups have reported that cordycepin has antiviral activity against several viruses including influenza virus, plant viruses, human immunodeficiency virus(HIV), murine leukemia virus, and Epstein-Barr virus (EBV). In this study, we identify the epigenetic mechanisms by which cordycepin exerts its anti-gammaherpesvirus effects. We show that cordycepin possesses antitumor and antiviral activity against gastric carcinoma and EBV, respectively. A comparison of the CD_50_ values of cordycepin and its analogs showed that the lack of a 2′-hydroxyl group in cordycepin was critical for its relatively potent cytotoxicity. Cordycepin treatment decreased the rate of early apoptosis in SNU719 cells by up to 64%, but increased late apoptosis/necrosis by up to 31%. Interestingly, cordycepin increased *BCL7A* methylation in SNU719 cells by up to 58% and decreased demethylation by up to 37%. Consistent with these changes in methylation, cordycepin treatment significantly downregulated most EBV genes tested. Under the same conditions, cordycepin significantly decreased the frequency of Q and F promoter usage, and H3K4me3 histone enrichment was significantly reduced at several important EBV genomic loci. Extracellular and intracellular EBV genome copy numbers were reduced by up to 55% and 30%, respectively, in response to 125 μM cordycepin treatment.

Finally, cordycepin significantly suppressed the transfer of EBV from LCL-EBV-GFP to AGS cells, indicating that EBV infection of gastric epithelial cells was inhibited. These results suggest that cordycepin has antiviral and antitumor activities against gammaherpesviruses and host cells latently infected with virus.

## INTRODUCTION

*Cordyceps*, a genus of ascomycete fungi, contains a fruiting body that grows on insects and other arthropods [1]. The genus includes *Cordyceps sinensis*, *Cordyceps militaris*, and *Cordyceps kyushuensis* [1]. *Cordyceps* species are known to produce many types of active components and are used for diverse medicinal purposes because of their varied physiological activities [2]. The active components include nucleic acid analogs (e.g., adenine, adenosine, cordycepin, and hypoxanthine), steroid compounds (e.g., 5α,8α-epidioxy-24(R)-methylcholesta-6,22-dien-3β-d-glucopyranoside, 5,6-epoxy-24(R)-methyl-cholesta-7,22-dien-3β-ol, 5α,8α-epoxy-24(R)-methylcholesta-6,22-dien-3β-d-glucopyranoside, 22,23-dihydroergosteryl-3-o-β-d-glucopyranoside, ergosteryl-3-o-β-d-glucopyranoside, 22,23-dihydroergosteryl-3-o-β-d-glucopyranoside, and ergosteryl-3-o-d-glucopyranoside), polysaccharides, and proteins [3].

The physiological activities include protection of liver damage as well as anticancer, antidepressant, anti-inflammatory, hypoglycemic, and antimicrobial effects [4]. In a recent study, *Cordyceps* water extracts were shown to activate Kupffer cells in the liver and prevent lung cancer cells from metastasizing to liver tissue [5]. In other studies, *Cordyceps* extracts were shown to enhance MHC class II antigen expression in liver cancer cells that contain low levels of MHV class II antigen, and *Cordyceps* polysaccarides inhibit proliferation of leukemia cells [6]. Currently, *Cordyceps* is commonly used as a health food supplement for the treatment of several disorders. However, further studies are required to develop *Cordyceps* into a viable therapeutic agent. The major physiologically active components produced by *Cordyceps* must be defined and the underlying molecular mechanisms of their actions need to be elucidated.

Cordycepin, an adenosine derivative, differs from adenosine in that its ribose lacks an oxygen atom in the 3′ position [7]. Cordycepin was initially isolated from Cordyceps, but is now produced synthetically. Because the chemical structures of cordycepin and adenosine are highly similar, some nucleotide polymerase cannot discriminate between cordycepin and adenosine. Consequently, cordycepin can be incorporated into an RNA molecule, thus prematurely terminating RNA synthesis [8]. Interestingly, cordycepin shows bioactive properties such as antitumor, antifungal, and antiviral activities that are attributable to its ability to inhibit several protein kinases [1].

Several research groups have reported that cordycepin has antiviral activity against a number of viruses including influenza virus [9], plant viruses [10], HIV [11], murine leukemia virus [12], and EBV [13]. High cordycepin concentrations selectively inhibit influenza viral genome replication [14]. Cordycepin analogs inhibit purified HIV-1 reverse transcriptase. *In vitro* RNA synthesis of tobacco mosaic virus and cowpea chlorotic mottle virus replicase are inhibited by high concentrations of cordycepin. Moreover, EBV-induced transformation of human lymphocytes is inhibited by cordycepin in the absence of interferons. Cordycepin analogs might inhibit transformation by affecting EBV mRNA, thereby preventing virus genome replication and subsequently causing defects in lymphocyte transformation.

As mentioned above, the molecular mechanisms by which cordycepin exerts its antiviral activities are yet to be determined. Recent studies of the underlying mechanism of cordycepin action has expanded on a replicase study, in which the negative effect of cordycepin on RNA synthesis were examined [8]. For example, Moor and colleagues reported that cordycepin inhibits protein synthesis and cell adhesion by affecting signal transduction [15]. Thus, we were prompted to clarify the molecular mechanisms that underlie the antiviral and antitumor effects of cordycepin. In this study, we define the epigenetic mechanisms by which cordycepin exerts its anti-EBV activity.

## RESULTS

### Cordycepin is cytotoxic to SNU719 cells

**Figure 1:**
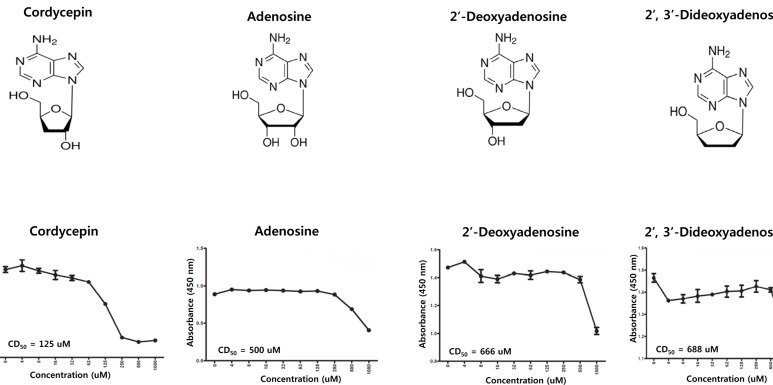
Cytotoxicity of cordycepin A cytotoxicity assay was performed using a cell counting assay (CCK-8 kit) to determine if cordycepin causes cytotoxicity. Cordycepin cytotoxic to SNU719 cells that latently infected by Epstein-Barr virus(EBV). The same methods were used to test the cytotoxicity of adenosine and its analogs. (A) Structures of cordycepin, adenosine, 2′-deoxyadenosine, and 2′,3′-dideoxyadenosine. (B) 50% cytotoxicity dose (CD_50_) of cordycepin and its analogs in SNU719 cells. Each measurement was repeated three times. Averages and standard errors of measurements are displayed on the graphs.

In order to determine the 50% cytotoxicity dose (CD_50_) of cordycepin and its analogs in SNU719 cells, cellular cytotoxicity assays were performed using CCK-8 (Figure 1A). The CD_50_ value of cordycepin in SNU719 cells was 125 μM, whereas those of adenosine, 2′-deoxyadenosine, and 2′,3′-dideoxyadenosine were 500, 666, and 688 μM, respectively (Figure 1B). Comparison of CD_50_ between cordycepin and its analogs indicated that lack of a 2′-hydroxyl group in cordycepin accounted for its stronger cytotoxicity compared to its analogs.

### Cordycepin treatment does not induce apoptosis or necrosis

**Figure 2:**
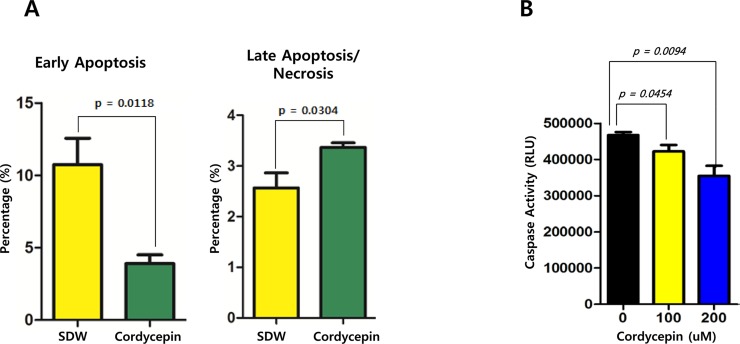
Effect of cordycepin on apoptosis Two apoptosis assays—Annexin V-FITC and caspase 3/7—were performed to analyze the effect of cordycepin on apoptosis in SNU719 cells. (A) The Annexin V-FITC apoptosis detection assay was performed in triple repeats. Early apoptosis was suppressed by cordycepin by up to 64%, but increased late apoptosis/necrosis by up to 31% in SNU719 cells treated with cordycepin for 48 h. SDW, blank treatment; Cordycepin, 125 μM cordycepin treatment. (B) The caspase 3/7 assay was conducted in triple repeats. Caspase 3 and 7 activities were gradually decreased in SNU719 cells in response to 100 and 200 μM cordycepin treatments. Numbers 0, 100, and 200 indicate the micromolar concentrations of the cordycepin treatments. P-values <0.05 (95% confidence) were considered statistically significant.

To elucidate the mechanism by which cordycepin causes cytotoxicity in SNU719 cells, an Annexin V-FITC apoptosis detection assay was used to analyze effects of cordycepin on early apoptosis and necrosis. Cordycepin treatment suppressed early apoptosis by up to 64%, but increased late apoptosis/necrosis by up to 31% in SNU719 cells (Figure 2A). A caspase 3/7 assay was also performed to confirm that cordycepin decreased early apoptosis in SNU719 cells. Cordycepin treatments at concentrations of 100 and 200 μM reduced apoptosis by up to 9.49% and 23.94%, respectively (Figure 2B). These results suggest that the cytotoxic effects of 125 μM cordycepin treatment were likely caused by cellular events other than apoptosis and necrosis.

### Cordycepin enhances cellular membrane integrity

**Figure 3:**
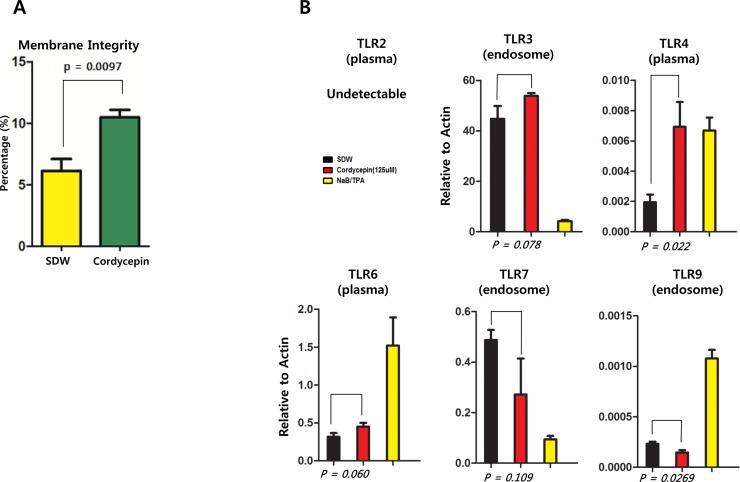
Effect of cordycepin on cellular membrane integrity The Annexin V-FITC apoptosis detection assay was used to determine if cordycepin affects SNU719 membrane integrity. A n RT-qPCR assay was then performed to determine if cordycepin affects membrane protein transcription. (A) Effect of cordycepin on SNU719 cell membrane integrity, as determined using a Annexin V-FITC apoptosis detection assay. As expected, cordycepin significantly enhanced membrane integrity in SNU719 cells. SDW, blank; Cordycepin, 125 μM cordycepin treatment. (B) RT-qPCR was performed to quantify TLR (Toll-like receptor) transcripts (*TLR2*, *TLR3*, *TLR4*, *TLR6*, *TLR7*, and TLR9) localized to cytoplasmic or endosomal membranes. Cordycepin enhanced *TLR4* and *TLR3* transcription, but downregulated that of *TLR7* and *TLR9*. *TLR2* transcript was not detected by this assay. SDW, blank; Cordycepin, 125 μM cordycepin; NaB/TPA, NaB (3 mM)/TPA (20 ng/ml) treatments. P-values <0.05 (95% confidence) were considered statistically significant.

Next, we tested whether plasma or endosomal membrane integrity was affected by cordycepin-induced cytotoxicity in SNU719 cells by using FITC Annexin V Apoptosis Detection Kit I. SNU719 cells were treated with 125 μM cordycepin for 48 h. Compared to cells treated with DMSO, SNU719 cell membrane integrity was increased by up to 71% (Figure 3A), indicating that cordycepin may induce membrane protein expression. To test whether cordycepin induced membrane protein expression, a reverse-transcription (RT)-qPCR assay was performed to quantify Toll-like receptor (TLR) transcripts such as *TLR2*, *TLR3*, *TLR4*, *TLR6*, *TLR7*, and *TLR9*. TLR2, TLR4, and TLR6 are plasma membrane-localized, whereas TLR3, TLR7, and TLR9 are localized to endosomal membranes. Cordycepin enhanced transcription of *TLR4* and *TLR3* (Figure 3B). In contrast, *TLR7* and *TLR9* were downregulated in response to cordycepin treatment, and TLR2 transcript was not detected using this assay (Figure 3B). These results indicate that cordycepin induces SNU719 cells to reinforce membrane integrity by increasing production of membrane proteins.

### Cordycepin does not affect cell cycle

**Figure 4:**
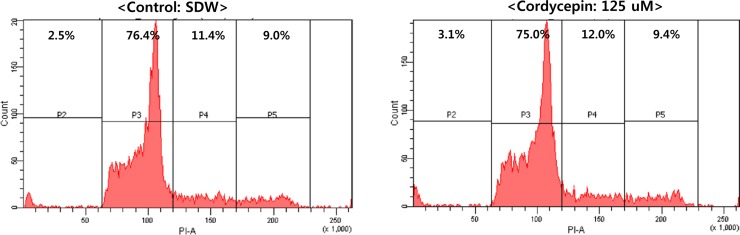
Effect of cordycepin on cell cycle Effect of cordycepin on cell cycle progression was examined using PI staining and FACs analysis. Cordycepin (125 μM) did not arrest SNU719 cells at any phase in the cell cycle.

To determine whether cordycepin affected cell cycle progression in SNU719 cells, PI staining and FACs analysis was performed on SNU719 cells treated with 125 μM cordycepin for 48 h. Compared to mock treatment (SDW), cordycepin treatment had no effect on SNU719 cell-cycle progression (Figure 4). These results suggested that 125 μM cordycepin treatment did not cause severe defects in cell cycle progression.

### Cordycepin induces methylation on genome locuses

**Figure 5:**
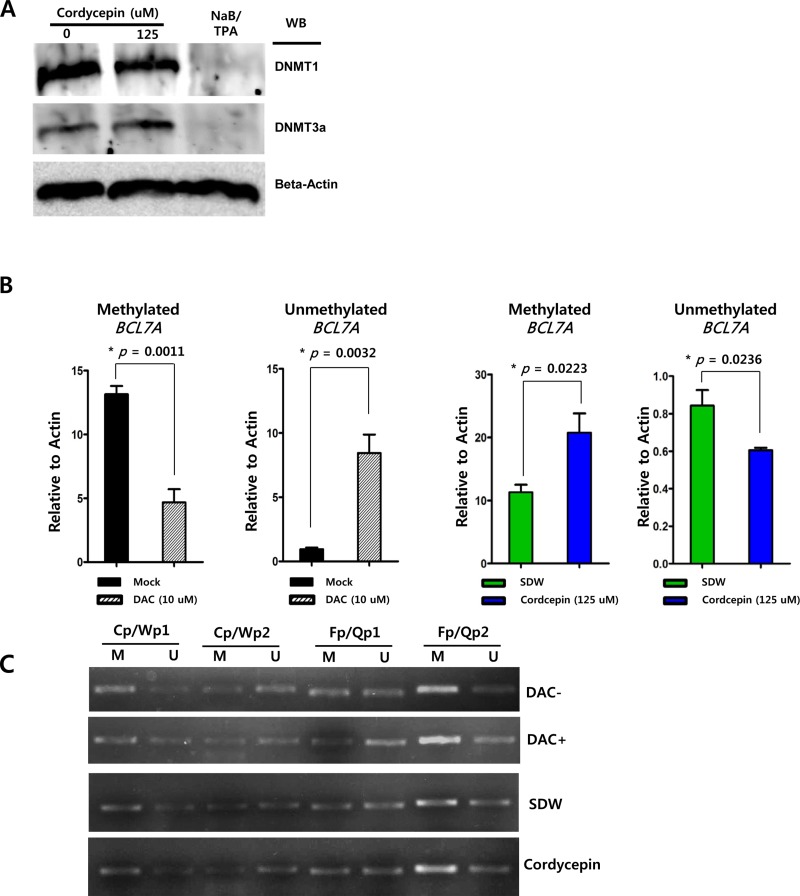
Effect of cordycepin on methylation The effect of cordycepin on methylation was determined using Western blot analysis as well as methylation-specific PCR and RT-qPCR assays. (A) Western blot analysis was performed to determine if cordycepin affected DNMT1 and DNMT3a production in SNU719 cells treated with cordycepin. Beta-actin was used as loading and internal controls. These Western blot analysis showed that cordycepin did not affect DNMT1 levels, but did significantly induced DMNT3 expression. (B) Methylation-specific PCR was performed on cordycepin-treated (125 μM) SNU719 cells to determine if cordycepin affected methylation of *BCL7A*, a tumor suppressor gene. Compared to the mock treatment, 10 μM 5-aza-2′-deoxycytidine (DAC) treatment suppressed methylation by up to 35% and induced demethylation by up to 882% (left two panels). The methylation-specific PCR assay showed that cordycepin treatment (125 μM) induced *BCL7A* methylation by up to 58% and suppressed demethylation by up to 37% in SUN719 cells (right two panels). P-values <0.05 (95% confidence) were considered statistically significant. (C) An RT-PCR assay was performed to determine if cordycepin affected methylation of EBV genomic loci in SNU719 cells treated with cordycepin (125 μM). Methylation of EBV genomic loci near Cp/Wp promoters was not affected by cordycepin treatment, whereas that around Fp/Qp promoters was enhanced. Specifically, regions downstream of the Fp/Qp promoters were highly methylated in response to cordycepin treatment. Cp/Wp1 and Cp/Wp2 are EBV genomic loci upstream and downstream of Cp/Wp promoters, respectively. Fp/Qp1 and Fp/Qp2 are EBV genomic loci upstream and downstream of Fp/Qp promoters, respectively. M, methylation-specific primers; U, demethylation-specific primers.

In order to determine if cordycepin affected methylation on CpG domains in cellular genes or EBV latent/lytic promoters, Western blot and methylation-specific PCR assays were performed. Western blot analysis performed using anti-DNMT1 and DNMT3 antibodies showed that cordycepin had no effect on DNMT1, but markedly affected DNMT3 levels (Figure 5A). Because SNU719 cells are known to be densely methylated at *TP73*, *BLU*, *FSD1*, *BCL7A* [23], *MARK1*, *SCRN1*, and *NKX3.1* loci [24], methylation-specific PCR following DNA bisulfite conversion was performed to determine whether cordycepin affected methylation of the tumor suppressor gene *BCL7A* and EBV promoter loci. Using this assay, we observed that cordycepin treatment increased *BCL7A* methylation by up to 58% and suppressed demethylation by up to 37% in SNU719 cells (Figure 5B). Furthermore, these data showed that cordycepin induced methylation at EBV genomic loci near Fp/Qp promoters (Figure 5C). Specifically, regions downstream of Fp/Qp promoters were highly methylated by cordycepin according to this assay. In contrast, methylation on both upstream and downstream regions of Cp/Wp promoters were not affected by cordycepin. These results suggest that cordycepin upregulates DNMT3, consequently enhancing methylation of genomic or EBV DNA loci in SNU719 cells.

### Cordycepin modifies cellular signal transduction

**Figure 6:**
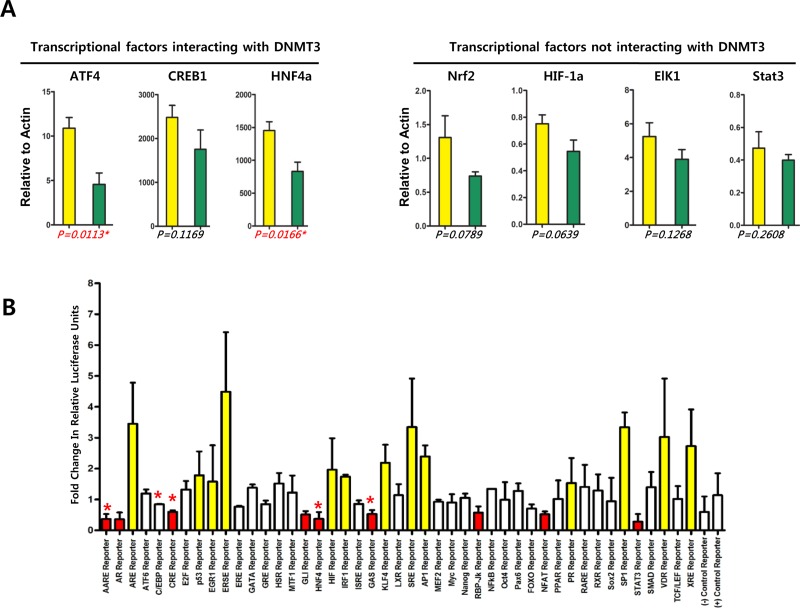
Effect of cordycepin on signal transduction A Cignal Finder reporter assay was performed to determine whether cordycepin-induced hypermethylation affects the expression of transcription factors that are known to interact with DNA methyltransferase (DNMT). An RT-qPCR assay was then performed to confirm the expression patterns of transcription factors that were upregulated or downregulated in the Cignal Finder reporter assay. (A) SNU719 cells were treated with cordycepin (125 μM) for 48 h and analyzed using an RT-qPCR assay. Transcription factors kwown not to interact with DNMT3 are *Nrf2* (ARE reporter, antioxidant response pathway), *YY1* (ERSE reporter, ER stress pathway), *HIF-1a* (HIF reporter, hypoxia pathway), and *Elk1* (SRE reporter, MAPK/EK pathway) [37]. These factors are downregulated by cordycepin, but this effect was not statistically significant. In contrast, transcription factors known to interact with DNMT3 are *ATF4* (AARE reporter, amino acid deprivation pathway), *CREB1* (CRE reporter, cAMP/PKA pathway) and *HNF4* (HNF4 reporter, HNF4 pathway) [37]. These factors are downregulated by cordycepin with statistical significance, especially the expression of *ATF4* and *HNF4*. P-values are listed at the bottom of the graph. (B) SNU719 cells were treated with cordycepin (125 μM) for 48 h and analyzed using the Cignal Finder reporter assay. Reporters induced by cordycepin are shown in yellow, and those suppressed by cordycepin are shown in red. AARE, AR, CRE, GLI, HNF4, GAS, RBP-jK, NFAT, and STAT3 reporters were downregulated by cordycepin. ARE, p53, EGR1, ERSE, HIF, IRF1, KLF4, SRE, AP1, PR, SP1, VDR, and XRE reporters were upregulated by cordycepin. Each reporter assay was performed in triplicate.

In order to define if DNTM3 induced by cordycepin downregulates transcriptional factors that are known to interact with DNMT3 [25], couples of interesting transcriptional factors were tested using RT-qPCR assay (Figure 6A). Among transcriptional factors known to interact with DNMT3, *ATF4* (AARE reporter, amino acid deprivation pathway) and *HNF4* (HNF4 reporter, HNF4 pathway) were significantly downregulated by cordycepin treatment though CREB1 (CRE reporter, cAMP/PKA pathway) was not significantly downregulated by cordycpein. On the other side, Nrf2 (ARE reporter, antioxidant response pathay), HIF-1a (HIF reporter, HIF-1a hypoxia pathway), Elk1 (SRE reporter, MAPK/ERK pathway), Stat3 (STAT3 reporter, STAT3 pathway) are known not to interact with DNMT3 and were not affected their transcription by cordycepin treatment. These factors appeared to be downregulated by cordycepin, but this effect was not statistically significant. To confirm downregulation of transcriptional factors by cordycepin, a Cignal Finder reporter assay was performed to determine whether cordycepin-induced hypermethylation affected the expression of transcription factors that are known to interact with DNMT3. Cordycepin treatment significantly downregulated 9 of the 45 transcription factors tested in SNU719 cells (Figure 6B). The following 9 transcription factors were downregulated: ATF4/ATF3/ATF2 (amino acid deprivation pathway), androgen receptor (androgen pathway), CREB1 (cAMP/PKA pathway), Gli (Hedgehog pathway), HNF4α (HNF4 pathway), STAT1 (Interferon gamma pathway), RBP-jK (Notch pathway), NFAT (PKC/Ca^2+^ pathway), and STAT3 (STAT3 pathway). Of these transcription factors, DNMT3 is known to interact with ATF4/ATF3/ATF2, CREB1 and HNF4α. Thus, we speculated that cordycepin-induced DNMT3 is involved in downregulating transcription factors by physically interacting with and methylating them. These results suggest that cordycepin likely suppresses the amino acid deprivation and HNF4 pathways by downregulating transcription factors such as ATF4/ATF3/ATF2 and HNF4α by specifically methylating their gene promoters. This suppression would eventually silence other transcription factors, EBV genes, and tumor suppressor genes.

### Cordycepin effects on EBV latent and lytic transcription

**Figure 7:**
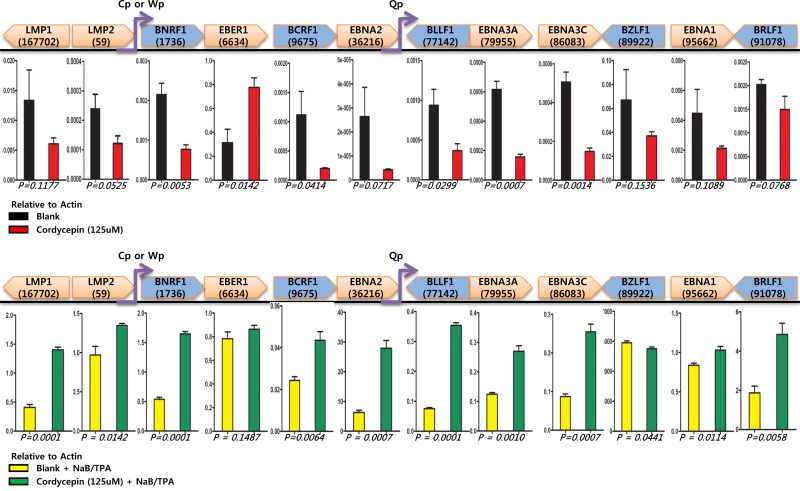
Effects of cordycepin on EBV latent and lytic transcription Effects of cordycepin on EBV gene transcription were determined by performing an RT-qPCR assay using cDNA from SNU719 treated with cordycepin. (A) cDNA synthesized from RNA isolated from cordycepin-treated SNU719 cells was analyzed using an RT-qPCR assay. The relative transcription levels of EBV latent and lytic genes was determined. Compared to mock treatment, cordycepin treatment increases only the transcription of the EBV non-coding RNA *EBER*. However, most EBV genes were significantly downregulated by cordycepin. Cp, Wp, and Qp are EBV promoters activated depending on the EBV latency type [17]. (B) cDNA synthesized from RNA isolated from SNU719 cells cotreated with cordycepin, sodium butyrate, and TPA was analyzed using an RT-qPCR assay. The levels of EBV latent and lytic genes transcription significantly increased. P-values <0.05 (95% confidence) were considered statistically significant. CTCF BSs, binding sites where CTCF are known to bind in the KSHV genome [22]. P-values are listed at the bottom of the graph.

To test whether cordycepin affects EBV transcription, we investigated transcriptional patterns in SNU719 cells treated with cordycepin by using a RT-qPCR assay. Compared to mock treatment, cordycepin treatment significantly increased transcription of the EBV non-coding RNA *EBER* (Figure 7A). *EBER* is known to be transcribed by RNA polymerase III, and was the most abundant RNA present in cells latently infected with EBV. In contrast, most EBV genes tested including *BLLF1* and *BcRF1* transcripts encoding glycoprotein gp350 and BcRF1, respectively, were significantly downregulated by cordycepin treatment (Figure 7A). However, cordycepin acted as a transcriptional activator when SNU719 cells were cotreated with HDAC inhibitors such as sodium butyrate and TPA (Figure 7B). Excluding *EBER1* and *BZLF1*, all genes tested were upregulated by cordycepin in cells cotreated with the HDAC inhibitors NaB and TPA (Figure 7B). These results indicate that cordycepin does not suppress EBV gene transcription by acting as a simple adenosine analog, because cordycepin also plays acts as a transcriptional activator.

### Cordycepin causes EBV to change the selection of latency promoters

**Figure 8:**
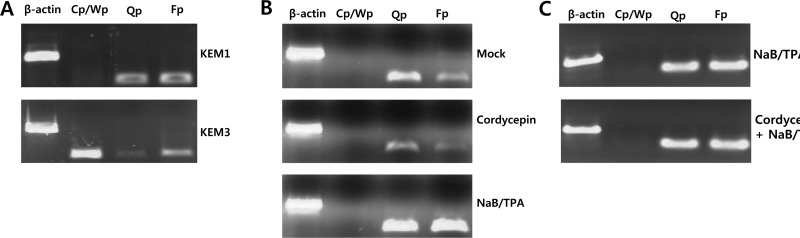
Effect of cordycepin on EBV latency promoter selection An RT-PCR assay was performed to determine if cordycepin affects the selection of EBV latency promoters. (A) As controls, KEM1 cells (specific to EBV type I) showed a high frequency of Qp and Fp promoter usage. KEM3 cells (specific to EBV type III) showed a high frequency of Cp/Wp promoter usage. (B) Cordycepin decreased frequencies of Qp and Fp promoter usage, but did not affect Cp/Wp promoter activity. (C) Combined treatment of cordycepin with HDAC inhibitors such as NaB and TPA did not reduce Qp and Fp promoter activities. Cp, Wp, Qp, and Fp are EBV promoters activated depending on the EBV latency type [17].

Because 125 μM cordycepin suppressed most EBV gene expression, we questioned whether cordycepin affected the selection of EBV latency promoters. As controls, KEM1 cells (EBV latency type 1-positive cells) showed a high frequency of Qp/Fp promoter usage (Figure 8A). KEM3 cells (EBV latency type 3-positive cells) showed a high frequency of Cp/Wp promoter usage (Figure 8A). Cordycepin significantly decreased the frequencies of Qp and Fp promoter usage (Figure 8B); however, cordycepin did not affect Cp/Wp promoter activity (Figure 8B). Moreover, cordycepin did not suppress Qp and Fp promoter activities in the presence of HDAC inhibitors NaB and TPA (Figure 8C). These results indicate that cordycepin suppressed Qp and Fp promoter usage to downregulate EBV genes.

### Cordycepin induces histone modification at EBV genomic loci

**Figure 9:**
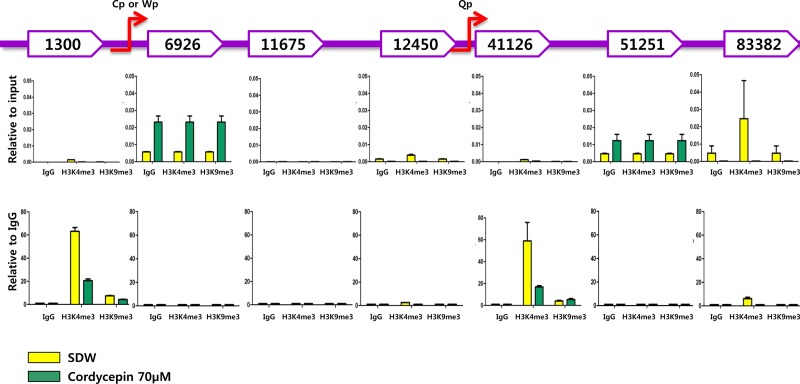
Effect of cordycepin on histone modification at EBV genomic loci Chromatin immunoprecipitation (ChIP) was performed to analyze EBV histone modification patterns in SNU719 cells treated with cordycepin. Seven EBV genomic loci in the EBV genome—1300 (*LMP2*), 6926 (between *BNRF1* and *BCRF1*), 11675 (between *BCRF1* and *BCRF2*), 12450 (5′ UTR of *BCRF2*), 41126 (between *BHLF1* and *BHRF1*), 51251 (*BPLF1*), and 83382 (*EBNA3B*)—were detected by RT-qPCR. Cordycepin reduces H3K4me3 histone enrichment at loci 1300, 12450, 41126, and 83382. Similarly, H3K9me3 histone enrichments at loci 1300, 12450, and 83382 were also a little reduced. These results indicate that cordycepin treatment results in histone modification at EBV genomic loci, consequently downregulating EBV genes.

Because cordycepin downregulated most EBV genes and decreased Qp and Fp promoter usage, we were interested in determining whether cordycepin affected histone modification at EBV genomic loci. Based on previous studies, we chose seven EBV genomic loci where H3K4me3 and H3K9me3 histones are enriched. These loci reside near EBV genome 1300 (*LMP2*), 6926 (between *BNRF1* and *BCRF1*), 11675 (between *BCRF1* and *BCRF2*), 12450 (5′ UTR of *BCRF2*), 41126 (between *BHLF1* and *BHRF1*), 51251 (*BPLF1*), and 83382 (*EBNA3B*) (Figure 9A and 9B) [26]. H3K4me3 histone enrichment was significantly reduced at loci 1300, 12450, 41126, and 83382 in response to cordycepin treatment (Figure 9A and 9B). Similarly, H3K9me3 histone enrichment at loci 1300, 12450, and 83382 (Figure 9A and 9B) also decreased due to the cordycepin treatment. These results indicate that cordycepin treatment results in histone modification at EBV genomic loci, consequently downregulating EBV genes.

### Cordycepin reduces EBV protein production

**Figure 10:**
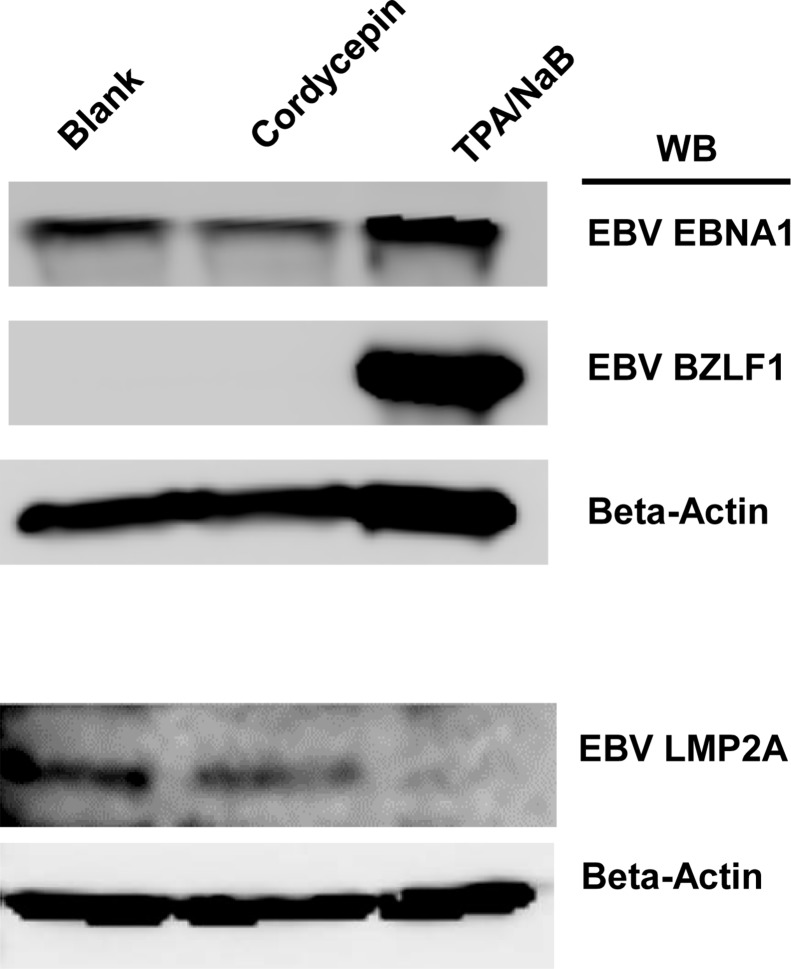
Effect of cordycepin on EBV protein production Protein levels were assessed by Western blot assay to determine the effect of cordycepin treatment on gammaherpesvirus translation in SNU719 cells. EBNA1 and LMP2A levels in SNU719 cells were greatly reduced, whereas BZLF1 expression was not changed by cordycepin treatment. Blank, Sterile distilled water(SDW); Cordycepin, 125 μM cordycepin treatment; NaB/TPA, NaB (1 mM)/TPA (1 ng/ml) treatment. β-actin was used as an internal control in the Western blot analysis.

Similar to the transcription assay, we were interested in testing whether EBV protein expression was affected by cordycepin treatment. To analyze EBV protein expression, a Western blot assay was performed using SNU719 cells treated with 125 μM cordycepin for 48 h. As observed for the transcription of viral genes, Western blot analysis showed that cordycepin significantly decreased EBV protein expression (Figure 10). Compared to mock treatments, cordycepin treatment greatly reduced EBNA1 and LMP2A levels in SNU719 cells, whereas BZLF1 expression levels were not changed (Figure 10). These results indicated that cordycepin treatment critically affected EBV translation and acts as a strong suppressor of EBV protein synthesis.

### Cordycepin decreases the EBV progeny production

**Figure 11:**
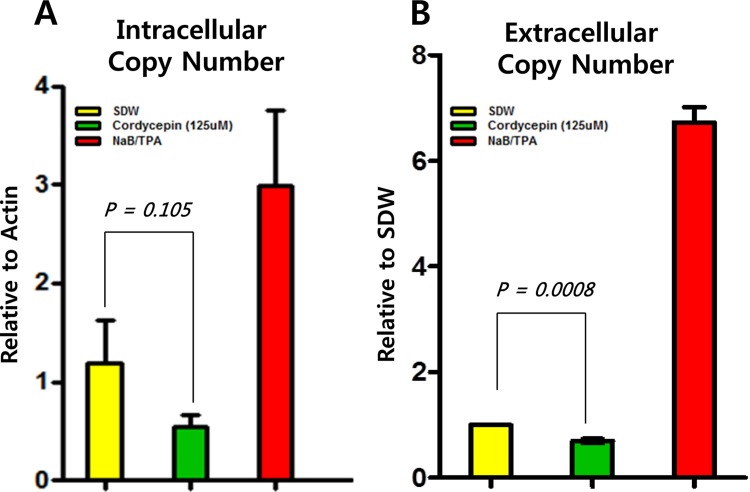
Effect of cordycepin on EBV progeny production Intracellular and extracellular EBV genome copy numbers were determined in SNU719 cells, following methods previously described. Intracellular (A) and extracellular (B) EBV genome copy numbers were significantly reduced by up to 55% and 30%, respectively, in response to 125 μM cordycepin treatment. SDW, blank; Cordycepin, 125 μM cordycepin treatment; NaB/TPA, NaB (1 mM)/TPA (1 ng/ml) treatment. Intracellular and extracellular are the relative intracellular (relative to GAPDH) and extracellular (relative to blank treatment) gammaherpesviral genome copy numbers, respectively. P-values <0.05 (95% confidence) were considered statistically significant.

Because most EBV genes tested were regulated by cordycepin, we next asked whether cordycepin stimulated EBV progeny production. To address this question, intracellular and extracellular EBV genome copy numbers were quantified from SNU719 cells by using methods previously described. Extracellular and intracellular EBV genome copy numbers were significantly reduced by up to 55% and 30%, respectively, after 125 μM cordycepin treatment (Figure 11A and 11B). These results indicate that cordycepin treatment suppresses EBV gene expression, which consequently decreases EBV progeny production.

### Cordycepin inhibits the EBV infection

**Figure 12:**
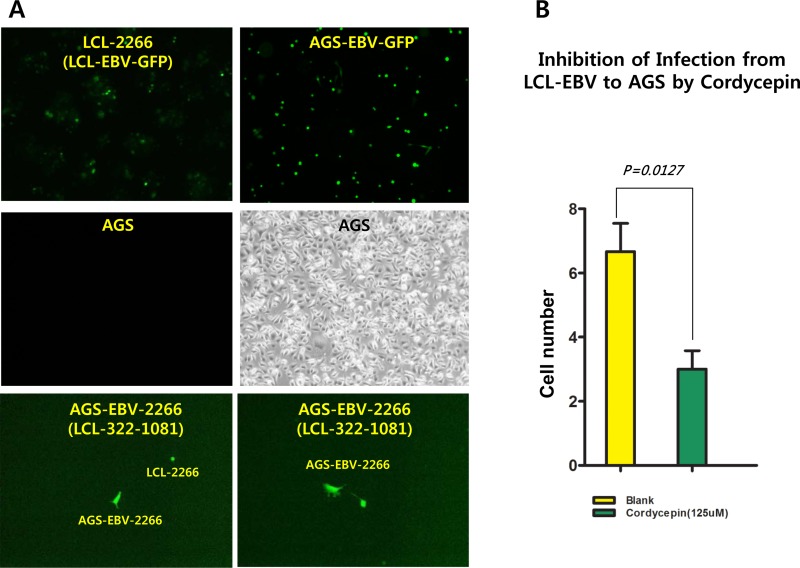
Effect of cordycepin on EBV infection A cell-to-cell coinfection assay was performed using LCL-EBV and AGS cells to determine whether cordycepin affects EBV infection. EBV transfers from LCL-EBV cells to AGS cells in the cell-to-cell coculture infection system [38]. As a donor, LCL-EBV-GFP cells (LCL-2266-36) were cocultured with AGS cells that had been seeded previously, and cordycepin treated was applied. (A) Cordycepin significantly suppresses the transfer of EBV from LCL-EBV-GFP cells to AGS cells, suggesting that EBV infection to gastric epithelial cells is significantly inhibited. (B) AGS cells infected with the EBV-GFP virus were attached and GFP-positive. P-values <0.05 (95% confidence) were considered statistically significant.

We asked whether cordycepin prevented transmission of EBV infection to virus-susceptible cells. An LCL-EBV to AGS cell-to-cell coinfection assay was designed to test if cordycepin inhibited the ability of EBV to infect AGS cells. EBV was transferred from LCL-EBV-GFP cells to AGS cells using the cell-to-cell coculture infection system (Figure 12A). Cordycepin significantly suppressed EBV transmission between these cell types, suggesting that EBV infection of gastric epithelial cells was significantly inhibited (Figure 12B).

## DISCUSSION

In this study, we showed that cordycepin has antitumor and antiviral activity against gastric carcinoma and EBV, respectively. First, a comparison of CD_50_ values of cordycepin and its analogs showed that the lack of a 2′-hydroxyl group in cordycepin results in stronger cytotoxic activity compared to its analogs (Figure 1B). Cordycepin treatment suppressed early apoptosis by up to 64%, but increased late apoptosis/necrosis by up to 31% in SNU719 cells (Figure 2A). Moreover, in comparison to DMSO treatment, cordycepin treatment increased SNU719 cell membrane integrity by up to 71% (Figure 3A). Interestingly, cordycepin increased *BCL7A* methylation by up to 58% and suppressed demethylation by up to 37% in SNU719 cells (Figure 5B). Methylation at EBV Fp/Qp loci was also significantly increased by cordycepin treatment (Figure 5C). Consistent with the methylation changes, cordycepin treatment significantly downregulated most of the EBV genes tested (Figure 7A), and only increased the transcription of the EBV non-coding RNA *EBER* (Figure 7A). Under the same conditions, cordycepin significantly decreased the frequencies of Qp and Fp promoter usage (Figure 8B), and H3K4me3 histone enrichment was significantly reduced at several important EBV genomic loci, including loci 1300, 12450, 41126, and 83382 (Figure 9A and 9B). Because EBV genes were downregulated, cordycepin treatment critically impacts EBV translation and acts as a strong suppressor of EBV protein synthesis (Figure 10). Extracellular and intracellular EBV genome copy numbers were significantly reduced by up to 55% and 30%, respectively, after 125 μM cordycepin treatment (Figure 11A and 11B). Finally, cordycepin significantly suppressed the transfer of EBV from LCL-EBV-GFP cells to AGS cells, suggesting EBV infection to gastric epithelial cells was inhibited significantly (Figure 12).

*EBER1*, a transcript produced by RNA polymerase III, is highly conserved among EBV strains [27]. Several proteins are known to bind to *EBER1*, including double-stranded RNA activated protein kinase (PKR) [28]. Downstream events of PKR signaling have been implicated in activating apoptosis [29]. In previous studies, *EBER1* was shown to inhibit IFNα-induced apoptosis by binding to PKR, which inhibits PRK autophosphorylation, consequently blocking downstream events involved in apoptosis activation [29]. The CD_50_value of cordycepin in SNU719 cells was 125 μM. This cytotoxicity was not due to apoptosis, but due to necrosis or late apoptosis in certain proportions. Apoptosis was actually decreased by cordycepin treatments that were shown to upregulate *EBER1*. Based on previous studies, we speculate that this suppression of apoptosis is possibly caused by cordycepin-induced *EBER1* upregulation. However, to dissect the exact molecular mechanism, further studies should be directed towards determining the effects of cordycepin on PKR signaling.

As DNA methylation at CpG dinucleotides near promoters efficiently represses gene transcription, it plays a crucial role in regulating the expression of cellular and viral genes during cancer development caused by tumor virus infection [30]. DNMT is known to cause gene hypermethylation despite an overall decrease in genomic cytosine methylation [31]. Interestingly, we showed that cordycepin markedly upregulated DNMT, especially DNMT3. *BCL7A*, a tumor suppressor gene, was found to be hypermethylated in response to cordycepin treatment. This methylation might be caused by DNMT3 hypermethylation. Under the same conditions, DNMT1 is known to interact with ATF4/ATF3/ATF2, CREB1, HNFα, and STAT1 [32]. These transcription factors were downregulated by cordycepin treatment at a concentration that increased DNMT3 expression. Thus, the interaction between DNMT3 and transcription factors might lead to the methylation of these factors, consequently decreasing their expression. Similarly, cordycepin likely induces DNMT3 expression, which might hypermethylate genes required to induce apoptosis, suppressing apoptotic cell death. Thus, DNA methylation at genomic loci is an important epigenetic mechanism used by cordycepin for antiviral and antitumor activities.

Three kinds of EBV latency types exist [33]; the Q promoter belongs to latency types I and II, while the C/W promoter belongs to latency type III. In contrast to latent expression, the F promoter is active when lytic genes are expressed. *BZLF1* and *BRLF1*, viral immediate–early lytic genes encode transcription factor Z (also known as ZTA and ZEBRA) and R (RTA), respectively. The proteins encoded by *BZLF1* and *BRLF1* activate one another, and amplify lytic-inducing effects, activating Z and R promoters [34]. Cordycepin likely suppresses EBV promoters and transcription factors, which might consequently downregulate EBV latent and lytic genes. However, interestingly, HDAC inhibitors switched the role of cordycepin from transcriptional suppressor to transcriptional activator. These results suggest that cordycepin does not play a simple role as an adenosine analog in suppressing EBV transcription. Instead, cordycepin has an important role as potent signaling molecule that might boost EBV gene expression by transducing physiological stimuli and chemical signals such as B-cell receptor(BCR) engagement, TGF-β, hypoxia, and DNA damage.

The eukaryotic genome is packed into chromatin, which comprises double-stranded DNA wrapped around core histone proteins [31]. Histone modifications such as methylation and acetylation play an important role in regulating gene expression by altering chromatin structure and condensation [35]. In addition to CpG methylation, several other epigenetic modifications can silence promoters including histone modifications, such as histone H3 lysine 27 trimethylation (H3K27me3), H4K20me3, or H3K9me3/me3 [36]. Among these repressive histone modifications, H3K9me3 is predominantly found in constitutive heterochromatin, and is regulated by enzymes including Suv39h [36]. Active markers such as H3K4me3, histone acetylation is associated with euchromatin. Cordycepin dramatically reduced H3K4me3 enrichment in near Qp and Cp promoter regions. Consistent with this loss of H3K4me3 enrichment, Qp promoter activity was significantly decreased by cordycepin treatment. EBV latent and lytic gene expression were reduced because of the reduction in Q (latency type I promoter) and F promoter (representative lytic gene promoter) activities. Similarly, intracellular and extracellular EBV genome copy numbers were decreased in response to cordycepin treatment. Thus, like DNA methylation, histone modification plays a key role in mechanisms underlying cordycepin activity.

In this study, we demonstrated that cordycepin has antiviral and antitumor activity against gammaherpesviruses and host cells latently infected with virus. Thus, *Cordyceps* can be used to protect host cells from both gammaherpesvirus infection and development of disorders caused by gammaherpesvirus infection. Future studies are warranted in order to develop *Cordyceps* into a medicinal food used for gastric carcinoma or lymphoma prevention. In this study, we clarified the molecular mechanism underlying the antiviral and antitumor activities of cordycepin, raising the possibility that *Cordyceps* can be developed as medicinal food to prevent gammaherpesvirus infection and resulting cancers.

## MATERIALS AND METHODS

### Cordycepin preparation

Cordycepin (purity: ≥98%) was obtained from Sigma-Aldrich Co. (St. Louis, MO, USA; catalog number C3394). Cordycepin was dissolved in sterile distilled water to make stock solution of 200 mM, which was then filtered through 0.22-μm filter (Sartorius Stedim Biotech) and stored at −20°C until use.

### Cell cultures

SNU719 cells, an EBV genome-integrated gastric carcinoma cell line [16], were cultured in RPMI1640 (Wellgene, Daegu, Korea) supplemented with 10% fetal bovine serum (FBS, Wellgene), antibiotics/antimycotics (Gibco, Grand Island, NY, USA), and Glutamax (Gibco) at 37°C, 5% CO_2_, and 95% humidity in a CO_2_ incubator.

### Reverse-transcription quantitative polymerase chain reaction

RNA was extracted from 125 μM cordycepin-treated SNU719 cells by using an RNeasy Mini Kit (Qiagen, Valenica, CA, USA), and was then synthesized into cDNA by using Superscript II Reverse Transcriptase (Invitrogen). The resultant cDNA was diluted 1:50 in nuclease-free water and used to analyze expression of EBV latent and lytic genes by quantitative polymerase chain reaction (qPCR). Primers for the following EBV latent genes were used: *LMP2A*, *EBER*, *EBNA2*, *EBNA3A*, and *EBNA1*. Primers for the following EBV lytic genes were used: *BNRF1*, *BLLF1*, *BZLF1*, *BRLF1*, and *BNLF2A*. Primers specific for transcription factor genes *Nrf2* (antioxidant response pathway), *YY1* (endoplasmic reticulum stress pathway), *HIF-1a* (hypoxia pathway), *Elk1* (MAPK/ERK pathway), *ATF4* (amino acid deprivation pathway), *CREB1* (cAMP/PKA pathway), *HNF4a* (HNF4 pathway), and *Stat3* (STAT3 pathway) were also used. Internal control gene primers were *GAPDH* and *GFP*. All primer set sequences have been published previously [17], and are available upon request. Positive controls used in these experiments was HDAC inhibitors such as sodium butyrate (3 mM) and 12-O-tetradecanoylphorbol-13-acetate (TPA, 20 ng/mL). qPCR was performed using iQ SYBR Green reagent (Bio-Rad) in qPCR CFX96 (Bio-Rad). Sample treated either cordycepin or mock were analyzed for EBV gene expression in triplicate. In addition, samples treated with either cordycepin and HDAC inhibitor or HDAC inhibitor only were also analyzed for EBV gene expression.

### Intracellular and extracellular EBV genomic DNA copy number quantification

Following lysis and sonication using a Bioruptor sonicator (Cosmobio, Tokyo, Japan; 5 min, 30-s on/off pulses), genomic DNA (gDNA) was extracted from 125 μM cordycepin-treated SNU719 cells. The resultant gDNA (50 ng) was subjected to qPCR analysis, and the relative amount of EBV gDNA was determined using the internal control *GAPDH*. Intracellular EBV copy number was calculated as the relative amount of EBV gDNA in the total gDNA. To determine the relative extracellular EBV copy number, 20-ml culture medium samples were collected from SNU719 cells treated with cordycepin. The culture medium samples were filtered through a 0.45-nm syringe filter, loaded onto a 20% sucrose cushion in phosphate-buffered saline (PBS) solution, and subjected to ultracentrifugation (CP100WX, Hitachi) at 27,000 rpm for 90 min. The virus pellet was lysed in 100 μl of FA lysis buffer [EDTA (1 mM, pH 8.0), HEPES-KOH (50 mM, pH 7.5), and NaCl (140 mM)], sonicated using a Bioruptor sonicator for 5 min (30-s on/off pulses), and DNA was extracted. Finally, viral DNA was resuspended in 100 μl of RNase-free water and qPCR analysis was used to quantify viral DNAs by using primer sets specific for EBV *EBNA1*.

### Cell cycle analysis

To assess the effect of cordycepin on cell cycle progression in SNU719 cells, they were treated with cordycepin, stained with propidium iodide (PI) solution for 48 h, and then subjected to cell cycle analysis by using a FACSAria III cell sorter (BD Bioscience; San Jose, CA, USA). Briefly, 3 × 10^6^ cells were seeded in 60-mm culture dishes. On the following day, when cell confluency reached 70%, SNU719 cells were treated with 125 μM cordycepin. The cells were harvested using trypsin at 48 h post-treatment, washed with cold PBS, fixed in 95% ethanol for at least 1 h, treated with 300 μg of RNase A, stained in 10× PI solution, and finally analyzed for cell cycle progress by using a FACSAria III cell sorter. Sterile distilled water(SDW) was used as mock in each treatment.

### Cytotoxicity assay

To evaluate the cytotoxic effects of cordycepin on SNU719 cells, a cellular cytotoxicity assay was performed using Cell Counting Kit-8 (CCK-8 ; Dojindo, Kumamoto, Japan) [18]. Briefly, 100 μl of cell suspension (1 × 10^4^ cells/well) was seeded and then treated with various concentrations (0-1000 μM) of cordycepin on the following day. After 48 h of cordycepin treatment, 10 μl of CCK-8 solution was added to each sample. Samples were incubated for another 3 h, and the absorbance of each cell suspension was then measured using an enzyme-linked immunosorbent assay reader. All steps followed the manufacturer's recommended protocol.

### Western blot analysis

To assess the effects of cordycepin on EBV protein synthesis, Western blotting was performed on SNU719 cells treated with 125 μM cordycepin. Treated SNU719 cells were harvested using trypsin at 48 h post-treatment. Cells (10 × 10^6^) were lysed using 100 μl of reporter lysis buffer (Promega) supplemented with 1 μl of proteinase inhibitor and 10 μl of phenylmethylsulfonyl fluoride. The cell lysates were further fractionated using the Bioruptor sonicator (5 min, 30-s on/off pulses). When necessary, the cell lysates were snap frozen in liquid nitrogen and stored at −80°C. Cell lysates (25 μl) were loaded onto 7% sodium dodecyl sulfate polyacrylamide electrophoresis gel and subjected to Western blot analysis by using antibodies against EBV proteins (1:1000 dilution). EBV EBNA1, EBV BZLF1, EBV LMP2A, DNMT1, and DNMT3 were detected, and β-actin was used as an internal control. Horseradish peroxidase-conjugated goat and sheep anti-mouse IgG were used as secondary antibodies.

### Apoptosis and membrane integrity analysis

To determine whether cordycepin induced apoptosis, caspase 3/7 and Annexin V-FITC apoptosis detection assays were performed on SNU719 cells that were treated with 125 μM cordycepin for 48 h. Caspase 3/7 activities in SNU719 cells were measured at 48 h post-treatment by using the Caspase Glo 3/7 kit (Promega, Madison, WI, USA). Briefly, 100 μl of cell suspension (1 × 10^4^ cells/well) was seeded and treated with 125 μM cordycepin the following day. Forty-eight hours post-treatment, Caspase Glo 3/7 reagent was equilibrated to room temperature prior to adding 100 μl of reagent to each well. After 3 h of incubation, cell suspension luminescence was measured at 560 nm. Apoptosis in cordycepin-treated SNU719 cells was also analyzed using an Annexin V-FITC apoptosis detection assay. Briefly, 5 ml of SNU719 cells (1 × 10^6^) was seeded in a 6-cm plate, and treated with 125 μM cordycepin (CD_50_ value) on the following day. After 48 h of treatment, cells were stained using FITC Annexin V Apoptosis Detection Kit I (BD Pharmingen, San Jose, CA, USA), and were then analyzed using a FACSAria III cell sorter 1 h post-staining.

### Methylation-specific PCR

To determine if cordycepin affects tumor suppressor gene methylation in SNU719 cells, a methylation-specific PCR assay was performed using DNA subjected to bisulfite conversion. Following lysis and sonication by using a Bioruptor sonicator (5 min, 30 s on/off pulses), gDNA was extracted from SNU719 cells treated with 125 μM cordycepin. We used the CpGenome DNA Modification Kit (Millipore, Billerica, MA, USA) for sodium bisulphite conversion of the DNA. The sequences of the *BCL7A* primers used are shown in Table 1. Each 25-μl reaction contained 5 μl of bisulfite-treated DNA template, 5 μl of 5× reaction mix (NanoHelix), 5 μl of 5× TuneUp solution (NanoHelix), 1 μl of Taq-plus polymerase (NanoHelix), and 2.5 μl of 10 μM forward/reverse primer. Primers were specific for methylated and unmethylated *BCL7A*. The primer pairs specific for regions upstream and downstream of Cp and Wp (Cp/Wp) as well as upstream and downstream of Fp and Qp (Fp/Qp) promotors are listed in Table 1. The following cycle conditions were used: 95°C for 3 min; 30 cycles of 95°C for 30 s, 55°C for 30 s, and 72°C for 30 s; followed by 72°C for 10 min. The reactions were performed using a TaKaRa PCR Thermal Cycler and then run on a 1.5% agarose/TBE gel.

**Table 1:** 

Genomic DNA		Primer sequence (5′- 3′)		Primer sequence (5′- 3′)
*BCL7A*	MF	GGTAGGCGACGTTTTAGTTC	UF	TGGGGTAGGTGATGTTTTAGTTT
	MR	GAATTAAAAACACCGATTCG	UR	CCAAATTAAAAACACCAATTCAA
Upstream of Cp/Wp region	MF	TTTAAAGTGGTAATAATATTAGGCGG	UF	TTAAAGTGGTAATAATATTAGGTGG
	MR	CTACATTTTTCAAATCGTAAACGAA	UR	CTACATTTTTCAAATCATAAACAAA
Downstream of Cp/Wp region	MF	GTTTTTTAGAGGAATTAGGGATTTC	UF	TTTTTTAGAGGAATTAGGGATTTTG
	MR	TCAAACATTCTTTAAATTTAACGAA	UR	CCCTCAAACATTCTTTAAATTTAACA
Upstream of Fp/Qp region	MF	TTTGGGGTATGGTATATTTAGTAGC	UF	TGGGGTATGGTATATTTAGTAGTGT
	MR	AACCTAATTCTTAACTCGTTCGAC	UF	AAACCTAATTCTTAACTCATTCAAC
Downstream of Fp/Qp region	MF	ATTGTTTTATTTAGTTGGTGGTGTC	UF	TGTTTTATTTAGTTGGTGGTGTTGA
	MR	CAAAATTTCCTAACTTTTTACGAA	UF	ACAAAATTTCCTAACTTTTTACAAA

### Cignal Finder reporter array

To determine what SNU719 cell signaling pathways were affected by cordycepin treatment, a Cignal Finder Multi-Pathway Reporter Array (Qiagen, catalog number CCA-901L) was performed. Attractene (1 μl/well) was distributed into a 96-well Cignal Finder Multi-Pathway Reporter Array plate. SNU719 cell suspension was diluted in Opti-MEM medium and then seeded in each well. Cells were treated with 125 μM cordycepin on the following day. After 48 h of treatment, reverse transfection reagent and Opti-MEM medium were removed. Dual-Glo Luciferase Reagent (75 μl) was then added to each well and plates were incubated for 10 min at room temperature. Finally, firefly and *Renilla* luciferase activities were measured following the manufacturer's recommendations. Briefly, 75 μl of firefly luciferase reagent was added to each well and then luciferase activity was measured. Afterwards, 75 μl of Dual-Glo Stop&Glo luciferase reagent was added to each well and *Renilla* luciferase activity was measured.

### Promoter usage detection assay

To determine if cordycepin affects the selection of EBV promoter usage, we performed conventional PCR on cDNA isolated from SNU719 cells treated with 125 μM cordycepin. Total RNA was extracted from SNU719 cells treated with cordycepin by using an RNeasy Mini Kit (Qiagen), and the RNA was then synthesized into cDNA using Superscript II Reverse Transcriptase. As controls, cDNA was synthesized from total RNA collected from KEM1 and KEM3 cells, which are EBV latency type 1 and 3 Burkitt lymphoma cells, respectively [19]. Primer sequences, including those for actin, EBV Qp, EBV Cp/Wp, and EBV Fp, were published previously [17]. cDNA was amplified in 25-μl reactions containing 5 μl of 5× reaction mix, 5 μl of 5× TuneUp solution, 1 μl of Taq-plus polymerase, and 2.5 μl of 10 μM of forward/reverse primer. The following cycle conditions were used: 95°C for 3 min; 30 cycles of 95°C for 10 s, 55°C for 30 s, and 72°C for 10 min; followed by 72°C for 10 min. The reactions were performed using a TaKaRa PCR Thermal Cycler and then run on a 1.2% agarose/TBE gel.

### Gammaherpesvirus infection

To determine whether cordycepin affects EBV infection of AGS cells (gastric carcinoma), we performed cell-to-cell coinfection by using AGS [20] and LCL-EBV-GFP B-cell lymphocyte cells [21]. AGS cells (0.625 × 10^6^/well) were seeded in 6-well plates (Corning, Corning, NY, USA). The following day, AGS cell culture medium—RPMI1640 (Hyclone, Logan, UT, USA) supplemented with 10% FBS (Hyclone), antibiotics/antimycotics, and Glutamax—was replaced with fresh medium, AGS cells were overlaid with LCL-EBV-GFP cells (1.25 × 10^6^/ml), and were treated with 125 μM cordycepin for 72 h. Seventy-two hours post-coinfection, we completely removed the medium and washed the cells twice with PBS, leaving only AGS-EBV-GFP cells. Bacto agar (1.5%) containing 2× RPMI1640 (Hyclone) supplemented with 20% FBS (Hyclone), antibiotics/antimycotics, and Glutamax was applied to the infected AGS-EBV-GFP cells. After 72 h of incubation, we counted GFP foci formed on AGS cells treated with either cordycepin or control treatment (sterile distilled water, SDW).

### Cross-linking chromatin immunoprecipitation

To analyze histone modification and methylation occurring in cordycepin-treated SNU719 cells, we performed cross-linking chromatin immunoprecipitation (X-ChIP) according the Upstate Biotechnology Inc. protocol and as described previously [22]. Genomic DNA was sonicated into 200- and 400-bp fragments by using a Bioruptor sonicator. Cell lysates were immunoprecipitated using H3K4me3 (Millipore, Billerica, MA, USA), H3K9me3 (Millipore), and IgG (Santa Cruz Biotechnology, Dallas, TX, USA; control) rabbit polyclonal antibodies. After immunoprecipitation, we decrosslinked the genomic targets of the DNA-binding proteins and then performed qPCR (BIO-RAD, Hercules, CA, USA; iQ^TM^ SYBR Green Supermix) to detect and quantify isolated gene fragments of interest. Primer pairs used in qPCR were designed based on previous publications that mentioned precise locations of histone enrichments and methylation (H3K4me3, H3K9me3) on EBV genome. These primer pairs are available upon request.

### Statistical analysis

Statistical tests were performed using the Student's t-test and ANOVA. P-values <0.05 (95% confidence) were considered statistically significant.
